# Obesity: Treatments, Conceptualizations, and Future Directions for a Growing Problem

**DOI:** 10.3390/biology11020160

**Published:** 2022-01-19

**Authors:** Julien S. Baker, Rashmi Supriya, Frédéric Dutheil, Yang Gao

**Affiliations:** 1 Centre for Health and Exercise Science Research, Department of Sport, Physical Education and Health, Hong Kong Baptist University, Kowloon Tong, Hong Kong 999077, China; jsbaker@hkbu.edu.hk (J.S.B.); gaoyang@hkbu.edu.hk (Y.G.); 2 CNRS, LaPSCo, Physiological and Psychosocial Stress, CHU Clermont-Ferrand, Occupational and Environmental Medicine, Université Clermont Auvergne, WittyFit, 63000 Clermont-Ferrand, France; fdutheil@chu-clermontferrand.fr

**Keywords:** obesity, prebiotic, probiotic, pharmacology, anti obesity drugs, future direction

## Abstract

**Simple Summary:**

The pyramid of interventions in the management of obesity include, diet restriction, diet therapy, physical exercise, anti-obesity drugs, prebiotics, probiotics, bariatric surgery, and cognitive behavioral strategies. Behavioral strategies that are being applied by predominantly private medical groups focusing on psychological assessments is appreciable; however, they are not replicated by all medical establishments, especially non-private health care institutions. This multifaceted disease has many treatment options; however, these options are not being fully implemented, especially in poor areas globally. Despite the upsurge in the market and expenditures in anti-obesity treatments currently available in economically stable areas of the world, obesity is still increasing at epidemic levels. The aim of this paper is to briefly highlight the anti-obesity treatments used presently and their inefficiency in isolation as individual treatments for obesity due to multifactorial causes. This report emphasizes that even focusing on psychological screening may not be enough to control this epidemic without further government and community cooperation. This report also presents novel strategies and radical thinking to include and amend successful interventions. Furthermore, we present details for incorporating web-based counseling and artificial intelligence to make treatments feasible, accessible, and cost-effective for all populations. We hope this paper will stimulate further debate and increase awareness in relation to the obesity problem and treatment strategies.

**Abstract:**

Interventions in obesity management include nutritional selection, diet restriction, and physical exercise, followed by cognitive behavioral strategies, pharmacology, and surgery towards the tapered treatment end of the obesity pyramid of interventions. Calorie restriction, regular exercise, and several weight reducing drugs, including probiotic and prebiotic use, are increasing in the market as potential anti-obesity treatments all over the world. Despite these efforts, obesity is increasing and is at epidemic levels. We propose here that there should be a multicomponent individual specific treatment approach for treating this multifactorial pathogenesis, incorporating psychological assessment as a first step that may help to reduce the prevalence of this alarming epidemic. We also believe that focusing on psychological screening may not be enough to control this epidemic without government and community cooperation and intervention. Additionally, we suggest that it is imperative to take advantage of the developments in web-based counseling and artificial intelligence expansion in combination with available anti-obesity treatments to make treatments feasible, accessible, and cost-effective for populations of all ages. The purpose of this paper is to increase awareness and stimulate debate in relation to this growing problem.

## 1. Introduction

The main aim of this report is to outline the inefficiencies in available obesity treatments. The obesity treatments discussed in this paper include dieting, exercise, probiotic/prebiotic supplementation, drugs, bariatric surgery, and behavioral approaches. We also discuss how the available obesity treatments are inefficient in isolation as an individual treatment of obesity. We further validate these problems by including examples from the United States. We highlight that anti-obesity treatments are increasing, yet the obesity epidemic is still prevalent. A secondary aim of the paper is to propose combined treatment strategies as current methodologies appear to be ineffective. We suggest that obesity needs a multicomponent approach with cooperation between private, public, and government sectors. This paper is fundamentally an opinion piece that will hopefully stimulate debate in the area.

## 2. An Overview of Obesity Treatment Options and Their Inefficiency in Isolation as Individual Treatments for Obesity

The pyramid of interventions for the management of obesity includes dieting, augmented by physical exercise, followed by cognitive behavioral strategies, pharmacology, and then surgery towards the tapered end of the treatment pyramid.

### 2.1. Dieting

Calorie restriction strategies are one of the most common dietary plans. A low-calorie diet refers to a diet with a total dietary calorie intake of 800–1500 calories, while a very low-calorie diet has less than 800 calories. Dieting aggressively lowers basal metabolic rate, which results in burning less energy at rest. This results in a significantly lower daily calorie requirement for maintaining weight after diet cessation. Normal eating habits at a reduced metabolic rate tend to encourage weight gain post-dieting. Physiologically, dieting is harmful, and the body readjusts for years after rapid weight loss to return to its original weight. In a recent analysis of 14 participants in the “Biggest Loser” contest, it was observed that contestants lost an average of 128 pounds and their resting metabolic rates decreased over a period of 30 weeks [1]. Metabolic rate decreased the most among those who lost the most weight. Almost all or most of the weight the contestants lost was regained by five of the fourteen contestants in the six years following the program, and their metabolic rates continued to be low despite the weight gain [1]. The metabolic adaptation caused by rapid weight loss is persistent over time, suggesting an incomplete but proportional response to contemporaneous attempts to reduce body weight. Food is treated as a reward or punishment in diets, and food obsessions can become problematic. When hunger is not satisfied, mood swings and overeating are likely to occur. Dehydration and further complications, such as constipation, can result when food is restricted despite drinking adequate fluids. Dieting and chronic hunger tend to exacerbate dysfunctional behaviors, such as smoking cigarettes, drinking alcohol, or substance abuse [1]. Dietary interventions have also been suggested as anti-obesity plans. Laura E. Matarese published a very informative paper in 2016 that provided information about dietary interventions, including macronutrients, micronutrients, and nutritional biochemistry, as a foundation for designing interventions [2]. The paper discussed how a metabolic rationale, including low-carbohydrate, ketogenic, or low-fat diets, can be applied to control obesity, as they can be implemented efficiently and result in weight loss [3,4]. A large majority of these recommended diets lack scientific integrity. Foods are composed of a variety of macronutrients, which make direct comparisons of different weight loss diets challenging because there is no precise definition of dietary prescription. Specific nutrients that have been found to act in a pharmacological manner to promote weight loss in addition to treating nutrient deficiencies have also been investigated. For instance, chromium (III) is biologically active and assists in carbohydrate and lipid metabolism. There is some evidence suggesting that chromium helps to regulate appetite, reduce carbohydrate cravings, and increase lean body mass. Furthermore, calcium and dairy products have also been proposed to regulate body weight without being thoroughly investigated, and a variety of hypotheses have been suggested to examine this proposal. To date, however, there is little evidence that dairy products or calcium supplements reduce body weight or fat mass. Another alternative calorie restriction strategy, intermittent fasting, has also been suggested. Studies have shown that intermittent fasting may have similar effects as calorie restriction [5] and may reduce the risk of diet-related diseases, such as metabolic syndrome [5,6]. Intermittent fasting may be beneficial for obesity, insulin resistance, dyslipidemia, hypertension, and inflammation [6]. Various claims regarding intermittent fasting have been made, although its long-term sustainability and benefits are unknown. The American Heart Association reported in 2017 that intermittent fasting may lead to weight loss, reduced insulin resistance, and a lower risk of cardiometabolic diseases [5]. In addition, certain foods produce inflammatory responses, including those foods with added sugar and processed meat. It is also accepted that several chronic diseases associated with inflammation may be dietary related. Nutrition and diet can affect inflammation, and according to Bahr and colleagues [7], dietary inflammatory indices can be used to identify, compare, and validate diets that are inflammatory across populations without requiring the use of biomarkers. This allows investigative associations between an anti-inflammatory diet and the risk of disease. We should therefore focus on long-term strategies that consider a variety of food options without unnecessary regulations, such that a healthy diet is incorporated as part of our “natural way of life”, which prerequisites expert consultation.

### 2.2. Exercise

In addition to diet manipulation, further benefits can also be gained from exercising regularly. Increasing the satiating effects of a fixed meal and enhancing the satiating effects of diet maximize the benefits of regular exercise for maintaining weight loss. The benefits of exercise in controlling obesity are numerous [8]. This is true regardless if a person has never exercised previously. Individuals who have not exercised previously may be unaware of how to perform certain exercises correctly, which could result in injury. Correct consultation with a physician prior to beginning an exercise program and consultation with a qualified trainer or coach to design a program tailored to an individual’s needs are prerequisites for embarking on an exercise regime. In addition, exercise suppresses appetite; however, many individuals use exercise as an excuse to overindulge in unhealthy foods. Individuals should be aware of the “halo effect”: when a person starts an exercise program, improper food consumption can derail all physical efforts. In a previous study, researchers studied 175 overweight, inactive adults by randomly assigning them to one of three different exercises: low intensity walking (12 miles per week), medium intensity jogging (12 miles per week), or high intensity jogging (20 miles per week). The volunteers were requested to maintain their usual diets during the study. The results from the study indicated that the subjects on the high-intensity exercise regimen had decreased abdominal fat, while those on the low- and medium-intensity regimens did not [9]. The findings suggest that it is important to consider both diet and the type of exercise regime suggested by the trainer or physician. Energy storage and body weight regulation mechanisms are finely tuned to preserve survival chances in response to variations of energy availability, as reflected by the metabolic flexibility of the system that adapts to both starvation and overeating. There are a number of metabolic disturbances that may occur if these mechanisms lose their flexibility as a result of calorie restriction or increased energy intake [10]. Researchers assessed the energy expenditures of participants who were overweight and obese after switching them from a high-carb diet to a low-carb ketogenic diet. According to the study findings, changing from a high-carbohydrate diet to a ketogenic diet significantly increased energy expenditure, measured using doubly labeled water. However, this was only evident on days when the subjects lived outside the metabolic chamber. This effect was not accounted for by increased physical activity, measured by accelerometry [11].

### 2.3. Probiotic/Prebiotic Supplementation

In addition to exercise and diet manipulation, medical and associated professions have also focused on gastrointestinal microbiota, probiotic bacteria (“probiotics”), and prebiotics to provide treatments for to the obesity problem. Probiotics are live microorganisms that, when consumed in sufficient quantities, provide health benefits to the host. Using specific bacteria from dietary ingestion with active antimicrobial activity results in enhanced barrier function and immunomodulation. This helps decrease the inflammatory response, and the use of these microorganisms may dramatically affect the management of obesity without the side effects associated with traditional pharmacotherapy. Strain-specific effects on body weight and metabolism related to the type of probiotic have also been reported. However, the identification of the strains that may have beneficial effects requires further research. As a result, their systematic use cannot yet be recommended in the treatment of obesity. Prebiotics in general terms are a food source for probiotics or beneficial bacteria in the digestive system. Essentially, prebiotics are carbohydrates that humans are unable to digest. These act as bifidogenic agents, improving the absorption of certain ions and trace elements while modulating cytokine expression. Both probiotic and prebiotic approaches comprise only a part of a weight loss strategy, and the dosage, administration duration, and long term consequences require further investigation [12]. It has also been recommended that probiotic/prebiotic interventions should start early in life to avoid obesity and the consequences of obesity during age progression. In addition, immune-compromised populations should be cautious when ingesting pre/probiotics because of the increased risk of infections associated with their use.

### 2.4. Drugs

In addition to diet, regular exercise, and pre- and probiotic use, several weight reducing drugs are available via prescription in consultation with medical professionals, while other less potent medications are available from websites and commercial outlets. Five drugs have been approved by the FDA—orlistat (Xenical, Alli), phentermine-topiramate (Qsymia), naltrexone-bupropion (Contrave), liraglutide (Saxenda), and semaglutide (Wegovy)—for long-term use [13]. Setmelanotide (IMCIVREE) is a sixth approved therapy that is restricted to people who have been diagnosed with one of three rare genetic disorders. Genetic testing is required to confirm the diagnosis. These medications may be taken provided the treatment is effective and there are no serious side effects. It has been reported that severe liver damage can result from taking anti-obesity pills. In many cases, multivitamins are prescribed daily to ensure that the individuals ingesting the pills are provided with enough vitamins, which they are unable to absorb from the food they consume. Patients with fluctuating heart rhythms, kidney disease, or mood issues, who are pregnant or thinking about becoming pregnant, breastfeeding, suffering from high blood pressure, seizures, or anorexia nervosa are advised to consult their health care provider prior to taking anti-obesity drugs. FDA-approved weight control medications that curb appetite are only approved for short-term use, typically for up to 12 weeks. Even though some health care professionals prescribe them for longer periods, very few studies have examined if they are safe and effective in the long term [13]. Epidemiological studies of anti-obesity medicines depict short-term success but long-term contraindications. The pharmaceutical industry seems to demonstrate exponential increases in withdrawn medications. The history of prescription medicines shows retrospectively that they have led to the death of millions and caused billions in compensation. The pharmaceutical industry currently seems to only be able to supply medicines that balance the equations relating to energy metabolism and weight control, thereby palliating the problem. Researchers are developing new medicines and manipulating the doses of old failed medications in an attempt combat the obesity pandemic. Even the latest dietary medicine “Semaglutide” recently approved by FDA in 2021 has limitations for certain populations. It is worth noting that Semaglutide plus a lifestyle intervention (not only ingesting the drug) was associated with clinically meaningful weight loss for overweight or obese people medicating with 2.4 mg once a week [14].

### 2.5. Bariatric Surgery

Apart from medicines, bariatric surgery has demonstrated beneficial effects in reducing body weight and controlling obesity related morbidities; however, the procedure is highly invasive and is only recommended to individuals with extreme obesity. Moreover, mild obesity combined with certain comorbidities, such as type II diabetes, can also be treated using this therapy. Bariatric surgery involves hormonal manipulation. This technique changes gut hormones due to a restriction and malabsorption associated with the procedure. In addition to promoting weight loss, reducing obesity-related comorbidities and mortality, and improving quality of life, bariatric surgery is recognized as a highly effective therapy for obesity. Overall, bariatric surgery reduces cardiovascular risk by 42% and all-cause mortality by 30%. However, bariatric patients may encounter some nutritional consequences that could compromise the benefits of this therapeutic option. As a result of bariatric procedures, patients can be more vulnerable to nutritional complications, namely, deficiencies of macro and micronutrients, which, in the case of anemia, osteoporosis, and protein malnutrition, can prove to be very serious. Several nutritional deficits are present in obese patients prior to surgery, the most significant of which are vitamin D and iron deficiency. As a result, a comprehensive nutritional evaluation and, eventually, an adequate correction of pre-existing deficits are necessary before surgery. Post-operative weight regain is also a significant issue following bariatric surgery and is commonly linked with obesity-related co-morbidities. The prevention of the nutritional complications associated with bariatric surgery can be achieved by life-long nutritional monitoring with the use of multivitamins and mineral supplements tailored specifically to each individual patient [15]. As new statistics reveal an alarming rate of obesity in the U.S., more people are opting for weight loss surgery to combat this disease. However, this treatment comes with a long list of possible side effects, including potential increases in the risk of suicide. It is important for doctors and patients to be aware of the potential psychological and physical side effects of bariatric surgery. Bariatric patients need a thorough psychological evaluation prior to surgery and careful follow-up post-surgery [16]. Moreover, according to perioperative guidelines, all patients should undertake a comprehensive mental health assessment prior to surgery [17].

### 2.6. Behavioral Approaches

Combining approaches in relation to obesity treatments has become increasingly important in recent years. Behavioral approaches are aspects being explored to treat obesity. In obesity treatment, behavioral interventions (or lifestyle modifications) are considered an essential cornerstone of treatment. With changes in diet and physical activity, these programs are designed to achieve long-term weight loss. Children and adults can both benefit from behavioral approaches for obesity prevention and treatment. In addition, they are fundamental components of surgical and pharmacological obesity treatments. The Weight Control and Diabetes Research Centre at The Miriam Hospital has conducted research on new behavioral weight control approaches [18]. Research participants in these studies receive free behavioral programs that are innovative in design. Diet interventions, physical activity, and behavioral strategies are part of a behavioral weight loss program. Calorie restriction targets provided individualized calorie deficits ranging from 500 to 1000 calories from baseline. Individuals weighing less than 200 pounds are prescribed a diet of 1000–1500 kcal/day. Those weighing more than 200 pounds are prescribed a diet of 1500–1800 kcal/day, and fat intake should be reduced to help achieve calorie goals. Exercise targets include moderate intensity activities, such as brisk walking, that begin with 50 min per week (10 min on five days of the week) and gradually increase to 150 min per week (30 min on five days of the week). There is a continuation approach to increase exercise time to 200 or 250 min per week if individuals want to maintain weight loss. As part of the behavioral strategy, participants are encouraged to increase their adherence to their diet and activity goals by self-monitoring (recording daily weight, diet, and activity), eliminating high-calorie foods from the home, setting goals, planning, and problem solving [19]. Programs for treating behavioral issues are generally offered in closed group settings, with about 15–20 patients attending weekly sessions for a period of 16–24 weeks. In the chronic disease model, the continuity of contact is important but will gradually be reduced to biweekly and monthly check-ins. Despite evidence that longer programs can increase weight loss and delay weight regain, they have been criticized as too expensive and burdensome to implement outside clinical settings. In recent studies, phone contact has been shown to be an effective substitute for face-to-face interaction. It is possible to disseminate these programs through mobile devices or the internet more cost-effectively [20]. A number of these approaches are derived from counseling methods used in psychotherapy for conditions such as smoking and alcohol misuse. As research reveals new information on the characteristics of weight loss, behavioral approaches are evolving. To alleviate the growing problem of the pandemic, we need to shift towards a patient-centered approach. In the United States for example, a preventive service for the treatment of obesity was implemented in November 2011 called ‘intensive behavioral therapy for patients’, and it includes face-to-face visits with a clinician organized by the Centre for Medicare and Medicaid Services [21]. Insurance companies have also replicated this service. Despite this development, clinicians face significant patient and clinician interactive challenges in the treatment of obesity. Patient-centered interaction is particularly difficult especially given the clinician’s time constraints in the clinic or office and medical priorities for patient treatments. The behavioral treatments available for weight loss have the most impact on weight loss maintenance, with some treatments sustaining 10% of weight loss after 36 months [21]. The gap between the subscribers who would be willing to pay for this low-cost intervention and the clinicians who would be qualified to deliver it is large enough to affect obesity in a positive way. Additionally, the incorporation of prebiotics and probiotics in the behavioral strategy must be considered.

We recommend here that the behavioral strategies that are currently being applied by certain private groups should focus primarily on psychological assessments and should also be incorporated into mainstream medicine with available access for all populations. We propose that prior to beginning any anti-obesity treatment plan, individuals should first undergo a psychological evaluation that allows for the identification of options and focuses specifically on the underlying causes of obesity. We suggest with a balanced diet, a good night’s sleep, regular exercise, and mindfulness practice, individuals can maintain a healthy body weight. Treatment pathways should be personalized and taken seriously and sympathetically, not only by the individuals who are overweight or obese themselves, but also by the clinicians and healthcare workers treating the patients. Initial steps should include the global development of accessible specialist obesity clinics. These clinics should provide specialist and individualized treatments from specialized staff, including clinicians, exercise scientists, nutritionists, psychologists, and consultants. This service should be available for all individuals regardless of their residential community or economic status. The treatment regimens should be available to all populations, including children and adults, and the clinics should be easily accessible and well-advertised. Strategies for treatment should include a specialist psychological assessment of the individual followed by physical and internal body assessment prior to prescription of any exercise, medicine, or surgery, along with behavioral, lifestyle, and any other supplemented intervention protocols.

## 3. An Introspective Dilemma: Anti-Obesity Treatment Supplies Causing the Obesity Boom?

**Figure 1:**
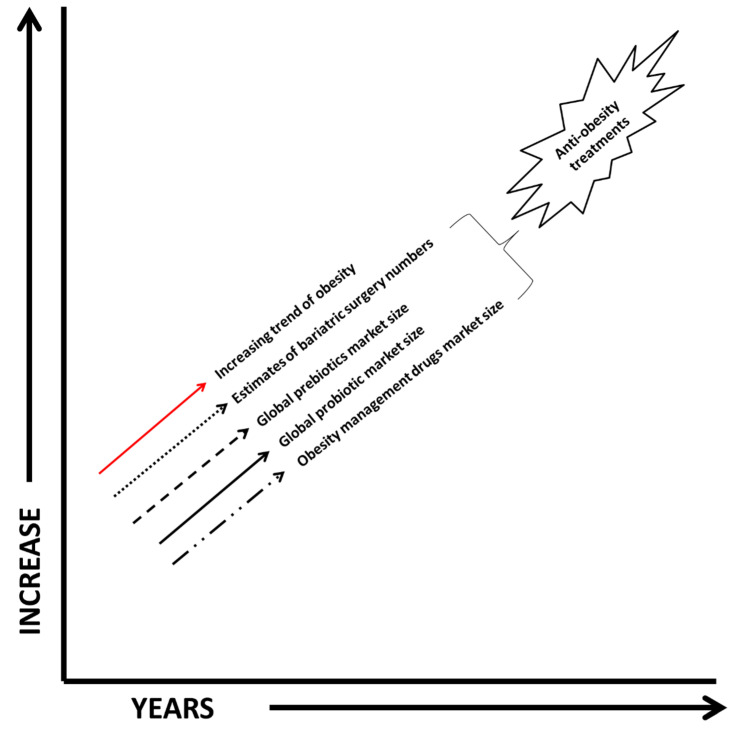
Ineffectiveness in obesity management despite various anti-obesity treatments [14,15,16,17,18].

The above-mentioned anti-obesity treatments and their developments (data available from US only) have been increasing consistently over time with an incredibly high economic burden on society (Figure 1). There has been limited success rates reported for the treatments; however, these procedures are unfortunately mostly performed in consultation with private medical provision. As a result, these remedies are not available to poorer sections of the community and underdeveloped countries. This is true in both western cultures and poor populations globally. Despite the upsurge in anti-obesity treatments in western cultures, obesity has been cited as a contributing factor to approximately 53,754–170,064 deaths in the United States per year and has increased health care use and expenditures, costing society an estimated $117 billion in direct (preventive, diagnostic, and treatment services related to weight) and indirect (absenteeism and loss of future earnings due to premature death) costs. The economic burden of obesity (estimated globally as US $990 billion per year) [22] is still increasing at an alarming rate in both children and adult populations (Figure 1). Hence, this multifactorial pathogenesis, including energy imbalance, insufficient lifestyle modifications, psychological issues, hormonal imbalance, and genetic and epigenetic factors, presents a substantial challenge to society and to the existing obesity treatment regimens which seem to be failing when using only one anti-obesity strategy in isolation.

We suggest that there should be a multicomponent individual-specific treatment approach for treating this multifactorial pathogenesis, incorporating psychological assessment as a first step that may help to reduce the prevalence of this alarming epidemic.

Despite the recommendations outlined above, neither therapy, including psychological screening, is particularly effective in isolation, and either individual therapy or a combination thereof may need to be continued indefinitely to protect against unavoidable weight regain. As discussed previously, anti-obesity treatments have some positive impacts but also some negative effects. Therefore, it seems clear that the problem of obesity must be managed and treated using a multicomponent approach involving behavioral therapy, psychological therapy, dietary changes and manipulations of gut microbiota, physical activity, and pharmacotherapy. It stands to reason then, that there needs to be a method for obesity management and a way to sustain effective programs. We believe a cost-effective solution to this epidemic can only be found if the public and private sectors combine and act in unison to fight the obesity problem.

## 4. A Growing Problem: New Thoughts!

### 4.1. How Can the Private Sector and Community Contribute?

The private sector, including workplaces and schools, should be proactive in facilitating anti-obesity measures. The evidence suggests that working more than forty hours a week can be associated with increased obesity, especially if the work environment is hostile [23]. The nature of work is changing; working hours are changing (both in terms of the duration and the frequency), the impact of technology at work, and psychosocial work factors affecting workers’ performance have an impact on obesity [24]. There have been several workplace weight management programs which have included education and counseling along with dietary and physical activity interventions. These studies have seen both short-term and long-term improvements in body weight profiles [25]. Similarly, to promote anti-obesity measures in the school environment, intervention programs that include exercise should be made mandatory for inclusion in the curriculum as a serious initiative to combat obesity. Anti-obesity measures should include dietary education and a description of the health problems and psychological issues associated with the onset of obesity. Among dietary interventions, school meal programs should include probiotics, prebiotics, and fiber-rich products in subsidized food menu options to promote better nutritional behaviors in school-age children nationally and internationally.

**Figure 2:**
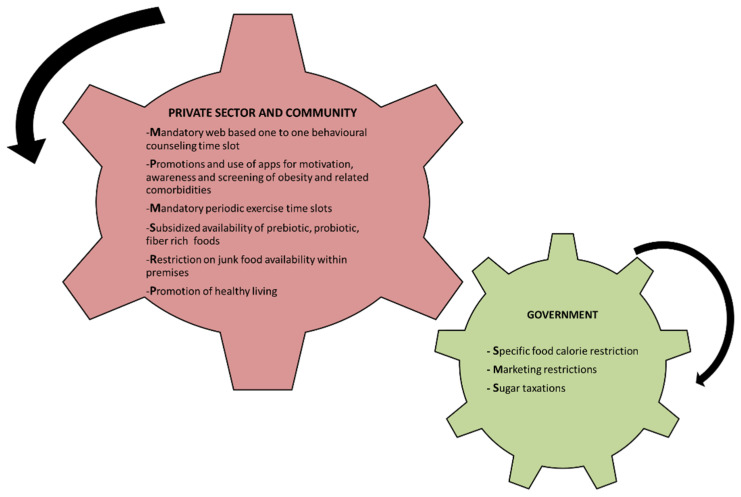
Suggested coordinated interactions between government, community, and the private sector.

Financial and manpower constraints pose the most significant barrier to the development and maintenance of weight management programs. A mandatory time slot should be created in every working organization for web-based one-on-one counseling. This has been suggested as an effective modern tool for managing obesity virtually [26]. Artificial intelligence (AI), which is part of the computer science field, could be effectively used to manage obesity and to minimize the threat of this disease. A recent review suggested that certain AI systems can be used in obesity management. These include the MOPET app to motivate physical activity; the decision support system for bariatric surgery patients; parameter decreasing methods and artificial neural networks to correlate obesity with cardiovascular disease; a neuro-fuzzy model to refine body mass index results; artificial neural networks to predict resting energy expenditure; a support vector machine that monitors food intake; and image processing algorithms. Research has concluded that all the AI systems investigated may have a tendency to produce more accurate results, suggesting that they are a potentially useful tool for managing obesity and related diseases [27]. We should be mindful that web-based obesity consultations and AI obesity management for children should be designed by incorporating game-like activities that children enjoy, and as a result will increase participation (Figure 2).

### 4.2. How Can the Government Contribute Further?

There are examples of government interventions in some countries that need reconsideration, such as specific food calorie reduction, marketing restrictions, and sugar taxation. Calorie reduction strategies are soft policies wherein the industry is encouraged to make changes based on government guidelines; however, these recommendations are not subject to fiscal regulation. Children are marketed for unhealthy food and drink consumption via a variety of platforms, including television, radio, movies, and games. A large percentage of marketing is used for advertising food and drink products with high fat, sugar, or salt content, which are associated with an increased risk of obesity and becoming overweight. The World Health Organization’s Global Strategy on Diet, Physical Activity and Health urged action against the marketing of unhealthy food and drink products in 2004. As a result, governments have been encouraged to support industry development of self-regulation, and to develop regulations to limit the advertising and marketing of nutrient-poor foods and beverages to children. Since 2007, food and drink advertisements are prohibited during, immediately before, and after programs targeted and primarily directed at or likely to appeal to children. As food and drink choices are largely determined by price, increasing costs can reduce purchases and consumption. Sugar taxes have been implemented in several countries across the world to reduce the consumption of sugary drinks, with promising results from Mexico, where a 7.6% decrease in the purchase of sugary drinks was noted over a two-year period [28] (Figure 2).

## 5. Conclusions

Ultimately, we conclude that this multifaceted disease has many treatment options; however, the treatments overall are unsuccessful. Treatment options require a multifaceted approach where psychological screening will help to narrow down the numerous options available to treat obesity. We also observe that poor populations and economically compromised individuals have limited access to the available treatments. Even focusing on psychological screening will not be enough to control this epidemic without sustained and meaningful government and community cooperation. In addition, it seems imperative that we focus on web-based counseling and artificial intelligence to make the treatment of obesity feasible and cost-effective for all individuals, regardless of their economic status. Therefore, this report highlights novel strategies and proposes the combination of amended past successful interventions to deal with this multifactorial disease. Further to this, we suggest incorporating web-based counseling and artificial intelligence to make treatment feasible and cost-effective for all individuals and populations of all ages. We hope this paper will raise awareness and stimulate debate among governments, policy makers, and medical professionals in relation to this increasing and alarming problem.

## Data Availability

Not applicable.
